# Conclusion and Recommendations: Implications and Way Forward

**DOI:** 10.1007/978-981-15-7018-6_40

**Published:** 2020-06-23

**Authors:** Brajesh Panth

**Affiliations:** 20 Education Sector Group, Asian Development Bank, Metro Manila, Philippines; 21 School of Education, RMIT University, Melbourne, VIC Australia; Education Sector Group, Asian Development Bank, Metro Manila, Philippines

## Abstract

The Asia and Pacific Region has made impressive gains in education and schooling in the past 50 years.

## Key Achievements

The Asia and Pacific region has made impressive gains in education and schooling in the past 50 years.^1^ The mean years of schooling for population 20–24 years increased from 3.5 years in 1960 to 8.9 years in 2010. Enrollments have increased at all levels of education with a much better gender balance. Almost all countries have achieved universal or near-universal primary education, while many have also achieved universal or near-universal secondary education. Many countries have also significantly expanded their technical and vocational education and training (TVET) and higher education.^2^ The performance of participating economies in the Programme for International Student Assessment (PISA) shows that some of the best education systems in the world are in the Asia and Pacific region, such as in Japan, the Republic of Korea, and Singapore.^3^ In recent years, while some participating cities in the People’s Republic of China are among the best performers, Viet Nam is widely viewed as being the best-performing lower middle-income country in the world. Many of these achievements have been possible largely due to public policy and investment but also due to the heavy involvement of the private sector which remains a major player in many countries, particularly in post-basic education.

## Major Challenges

Despite remarkable progress in improving access at all levels of education, developing Asia is facing four major challenges. First, most of the worlds’ 15–24 years youth with low reading proficiency are in the Asia and Pacific region, and a significant percent of 10-year-olds attending schools in the region cannot read a simple story. These learning deficits are so serious that there is a “ learning crisis.”^4^ Second, the region is facing serious skills mismatches.^5^ In some cases, a majority of education and training providers indicate that they are providing the skills needed by the employers, while the employers indicate that only around one-third of job applicants meet their job requirements.^6^ Third, many Asian countries are underinvesting in education. While UNESCO recommends that countries need to spend around 4–6% of their gross domestic product, or 15–20% of the annual budget, on education to achieve Sustainable Development Goal 4 by 2030, many countries in the region are spending less than this recommended level, making it difficult to make good progress by 2030. Along with an adequate share of the budget, the quality of expenditure to ensure that resources are prioritized on proven strategies is equally important. Finally, many countries lack a capacity to undertake and sustain reforms that require new, innovative thinking, and new approaches to equip learners with twenty-first- century skills and to forge effective new partnerships to sustain reforms.

## Moving Forward

As noted in the introductory chapter, and as implied throughout this book, education systems that were founded 100–150 years ago to meet the needs of the first and second industrial revolutions (which is the case for most education systems in countries throughout the world) are failing to meet the needs of the Fourth Industrial Revolution (4IR) and twenty-first-century skills that go beyond the three Rs (reading, writing, and arithmetic) to include digital and soft skills. Against this backdrop, the book has attempted to provide the following messages.

**1. It is important to rethink and reimagine education and training (Part I)**. At a time when technological change and automation are disrupting virtually all spheres of daily life, there is a need to rethink and reimagine education and training to make sure it meets the needs of all types of students, to prepare them for current, emerging, and future skills and jobs. The risk of proceeding with a “business as usual” approach is likely to exacerbate inequity since those that have access to twenty-first-century skills will have a much greater edge over those without such opportunities.

Research referred to in this book clearly shows that, more than school attainment, the quality of schooling is more closely associated with economic growth (Hanushek, Chap. 10.1007/978-981-15-7018-6_4). This relationship is a more powerful indicator of how investments in human capital can serve as a robust predictor of nations’ ability to sustain economic development and innovate. A two-pronged strategy is necessary for developing countries to “ go back to the basics” to improve learning outcomes for all students while paving opportunities for leapfrogging. It is not only important to ensure *learning for all* in schools but also to ensure that they are ready to meet the changing needs of today’s job market, is key (Lee, Chap. 10.1007/978-981-15-7018-6_5). Education systems need to produce self-directed learners because learners need the right skills and they should also know how to apply such skills as a foundation for sustainable development (Schleicher, Chap. 10.1007/978-981-15-7018-6_7). Due to rapid advancements in education technologies, there is a real opportunity to leverage such technologies, especially through online and blended learning, to improve teaching and learning at scale (Kim, Chap. 10.1007/978-981-15-7018-6_3).

While the mean years of schooling have increased in all developing countries, they still have a small share of people in the workforce with higher level skills. Ultimately, for developing countries to accelerate development, they will need to “leapfrog industrialization to the high-tech economy” and this will require prioritizing investments in people for higher level skills (Frey, Chap. 10.1007/978-981-15-7018-6_2). This is consistent with labor market projections around the globe which indicates that there will be less demand for lower level skills and more demand for higher level skills in the coming years. To develop such skills, universities have to transform and enable “academic entrepreneurs in close partnerships with industry” to provide a mix of hard and soft skills including entrepreneurship skills to innovate (Koh, Chap. 10.1007/978-981-15-7018-6_6).

**2. Schools need to customize rather than standardize education to meet the learning needs of all types of learners (Part II).** The main criticism of the current school system, which has little changed over the past 100–150 years, is that it treats all students as one assembly-line product. But there is an increasing recognition that a “ one-size fits all” approach is leaving the majority of learners behind since they come with diverse learning needs and backgrounds. According to the multiple intelligence theory, all students have the potential to learn and excel, but teaching and learning need to cater to the different learning styles of learners—some learners are logically mathematically oriented, some are verbally oriented, and some are physically oriented.^7^ Similarly, some are fast learners and some are slow learners. This means that current education systems have to transform by moving away from classifying all learners into one category—as average learners.^8^ Brain science research asserts this line of thinking and that teaching and learning have to be adapted to the needs of learners. In the absence of continuous formative evaluation, and understanding the learning needs of different students, many teachers in developing countries are unable to teach at the grade level.

The biggest challenge for most low-income and middle-income countries is the learning crisis that they are facing, despite remarkable progress in access to education. In their blog on learning deficits, the director of UNESCO’s Global Monitoring Report, and the director of the UNESCO Institute for Statistics, have cautioned that “without a shift from ‘business as usual’, the world will miss its goal of a quality education for all by 2030.”^9^ Their concern is based on their projections on progress countries are making toward Sustainable Development Goal 4. Similarly, a report by the International Commission on Financing Global Education Opportunity notes that “if current trends continue, by 2030 just four out of 10 children of school age in low- and middle-income countries will be on track to gain basic secondary-level skills.”^10^

 Rote learning is rampant in most developing countries and, in many cases, it has been difficult for teachers to apply a learner-centric pedagogy. Some evaluation of teacher professional development shows that such training is not leading to improvements in learning outcomes. National student assessments show that over 50% of 10-year-olds cannot read a simple story.^11^ For many developing countries that have participated in PISA tests, their poor performance is calling for a rethinking of their approach. “Boosting student learning starts with a good understanding of challenges that countries face, and best practices that could be learnt from the experiences of others” (Belfali, Chap. 10.1007/978-981-15-7018-6_8). For example, “Viet Nam has made excellent progress in developing a teacher assessment system based on national professional standards” and it aims to “unify that system with the professional development of teachers.” (Cammaert, Nguyen, and Tanaka, Chap. 10.1007/978-981-15-7018-6_12. Similarly, in the Philippines (Bernido and Bernido, Chap. 10.1007/978-981-15-7018-6_9) and Pakistan (Aziz, Chap. 10.1007/978-981-15-7018-6_11), nongovernment organizations are complementing the government’s efforts to provide good quality education. Building on such partnerships, and with the use of educational technologies, it is possible for teachers to use assessment tools to continuously monitor students’ learning levels and align classroom instruction to ensure that every child is effectively learning (Kumar, Chap. 10.1007/978-981-15-7018-6_10).

**3. Technical and vocational education and training requires public–private partnerships to respond to industry 4.0 needs (Part III)**. All governments in the region are concerned about quality jobs. Since rates of youth unemployment are usually 2–3 times higher than regular unemployment rates, governments are very keen to enhance the employability of the youth. However, the technical and vocational education and training (TVET) systems in many developing countries are supply-driven and fragmented due to the involvement of multiple agencies, and they are generally underfunded. Rapid changes in technologies, automation, unprecedented labor mobility, and aging populations are prompting governments to rethink workforce development. Labor market projections indicate that, due to increasing automation, lower order skills of a repetitive nature will be replaced by machines, while there will be a shortage of workers with higher order skills. This has huge implications for how education and training need to respond to the future of skills and jobs to reduce growing skills mismatches in an age of continuous digital disruptions and short shelf life of skills.

The dual training system that Germany, Austria, and Switzerland have successfully implemented is seen as one promising model for preparing job-ready workers. In the Philippines, the dual training program that the government is promoting under its flagship K-12 reform shows how the 6-month to 2-year certificate programs with technical support from GIZ are preparing job-ready learners through partnerships between employers (Philippine Chamber of Commerce), government (Technical Education and Skills Development Authority), and selected training providers (Don Bosco, German Confederation of Skilled Crafts and Small Businesses) (Dernbach, Chap. 10.1007/978-981-15-7018-6_13). In New Zealand, the industry training organizations are recognized for working with industries to develop occupational standards and training and assessment packages, as well as in promoting on-the-job training and school to work transitions (Williams, Chap. 10.1007/978-981-15-7018-6_15).

Countries with large shares of youth population realize the urgency to provide their youth with employability skills to reap the benefits of demographic dividends. This requires not only providing occupational skills but also soft skills since many youths may come with inadequate skills due to weak schooling. In India, where over 80% of the workforce is in the informal sector and there is a high attrition of skilled workers, Gram Tarang Employability Training Services, a social entrepreneurial partnership approach with Centurion University in Odisha, India is skilling and upskilling migrant workers from disadvantaged groups in close partnerships with industries to provide job-ready skills in sectors such as manufacturing, automotive, and hospitality (Madan, Chap. 10.1007/978-981-15-7018-6_16). In Bangladesh, another country with a large percentage of the youth population, the Ministry of Finance is directly engaging selected industry associations in priority growth sectors to skill and upskill the workforce with relevant education and skills to improve productivity and to address the emerging skills needs to be propelled by IR 4.0 (Lee, Chap. 10.1007/978-981-15-7018-6_17).

In the People’s Republic of China (PRC), where the population is aging fast, the Government is focusing on several complementary areas to strengthen the quantity and quality of TVET graduates. The Government has expanded the capacity of TVET at the senior secondary level by developing a sound legal framework that requires TVET trainers to have industry experience, promote work-based learning with quality assurance and credible assessment and certification, in close collaboration between industry and TVET institutions, and enhance the use of information and communications technologies in the delivery of TVET programs (Maruyama, Chap. 10.1007/978-981-15-7018-6_18). Since the PRC has been improving the quality of compulsory education up to grade 9, the improved foundational skills from better schooling provide a good foundation needed for TVET programs. Where students are coming from weak schooling, it is important to complement occupational skills with foundational skills.

**4. Higher education needs to be practically oriented to prepare graduates for higher level skills with an innovative and entrepreneurial mindset (Part IV).** Successful countries like the Republic of Korea (ROK) and Singapore have transitioned from “low skill equilibrium” to “high skill equilibrium” by building a solid school system followed by market-responsive TVET and a high-quality higher education system. These countries have demonstrated how they have evolved as economic powerhouses by investing in human capital despite lacking natural resources, and with the availability of cheap labor being short-lived. While other countries in the region are trying to emulate this success, for such a strategy to work well, it is important to consider different innovative models of universities to develop higher level skills and entrepreneurs and to spur innovation. In other words, high-quality universities can help leapfrog development by preparing high-tech graduates in priority areas such as modern agriculture, biotechnology, and information and technology.

The Hong Kong University of Science and Technology in Hong Kong, China, demonstrates how a well-funded university in a vibrant city led to high-quality science and technology and business programs by attracting world-class faculty, and how it adopted a unique entrepreneurial research culture that promoted transfer and the commercialization of technologies (Postiglione, Chap. 10.1007/978-981-15-7018-6_19). In the ROK, high-quality universities such as the Korea Advanced Institute of Science and Technology have contributed to the ROK’s transition into a knowledge-based economy by promoting science, technology, and entrepreneurship. In Indonesia, the Institut Teknologi Bandung is institutionalizing innovation and entrepreneurial spirit that has led to 70 start-ups and 6 spin-off campuses. ETH Zurich is yet another example of a high-quality university that has been a driving force of industrialization in Switzerland since its establishment in 1855.

The collaboration between Shenzhen Municipal Government and Tsinghua University provides another example of how the municipality has successfully evolved from a fishing village to a thriving commercial and innovation hub by attracting high-quality universities that have collaborated successfully with industries in promoting innovation and joint research and the commercialization of new products (Kang, Chap. 10.1007/978-981-15-7018-6_20). In the Pacific Region, the University of the South Pacific (USP) serves as a regional university, owned by the governments of 12 Pacific island countries, by adopting a regional cooperation strategy to support small island countries to provide good quality higher education through a network of branch campuses in all the member countries (Thonden, Chap. 10.1007/978-981-15-7018-6_21). By also linking with different networks (American, Australian, New Zealand), USP has been able to maintain its quality and achieve high recognition. Similarly, the State University of New York is fostering high-quality entrepreneurial ecosystem in the ROK through a Center of Global Entrepreneurship which was launched in 2017 in collaboration with a global and domestic network of entrepreneurship communities (Hsieh, Chap. 10.1007/978-981-15-7018-6_22).

**5. Education Technology has the potential of transforming teaching and learning and in providing twenty-first-century skills by supporting personalized learning and helping teachers to customize learning (Part V)**. Unlike in the past, education institutions are not the only place where students learn. Students come from different backgrounds with diverse learning styles and needs. At the school level, in addition to the 3Rs (reading, writing, and arithmetic), schools are also required to provide twenty-first-century skills such as soft, digital, and entrepreneurial skills. Developing countries that are struggling to attract qualified teachers with strong content knowledge are facing an additional challenge of ensuring learning for all. Given that many developing countries are facing a learning crisis, it will not be possible to make the desired progress in improving learning levels by 2030 without major transformative changes.

 EdTech solutions offer effective ways of transforming teaching and learning in a number of ways: (i) students can improve learning outcomes at their own pace and learning level through adaptive learning programs; (ii) rich contents can be drawn from global good practices and in partnership with employers in close alignment with national curriculum; (iii) teachers can continuously enhance their knowledge and skills through blended online teacher professional development programs; (iv) teachers can use EdTech programs to continuously assess student learning to align their teaching at the grade level; (v) teachers can use learner-friendly content to improve their instructions; and (vi) the governance and accountability of education systems can improve by sharing the performance of students, teachers and schools on a regular basis with key stakeholders.

With growing investments in EdTech solutions, the emerging trends show promising results. During the early stages of online learning, the emphasis was overshadowed by too much of a focus on hardware and an inadequate support on software and content. However, with time, there is growing evidence of EdTech programs having a positive impact on student learning outcomes (Garcia, Chap. 10.1007/978-981-15-7018-6_23).^12^ There are different examples of good practices in ICT-based learning that look promising: (i) there are several online and blended learning providers of massive open online courses that can be useful in ramping up skills in high demand courses such as machine learning, artificial intelligence, big data analytics, and coding and (ii) there are learning management systems that enhance governance and accountability of teaching and learning institutions (Pavlova, Chap. 10.1007/978-981-15-7018-6_24). While it is important to take calculated risks with the use of EdTech solutions, it is important to embed research with such programs to build evidence and demonstrate what works and what does not.

To address the needs of the 4IR, there is growing recognition among key stakeholders that the traditional education systems require total transformation. Mobile learning is increasingly promising given the easy access it provides to millions of learners in a flexible way and at a fraction of a cost for traditional education. The “ubiquity of digital software and mobile technologies have created a new set of rules, a new world order, that is unlike anything we have seen before” (Pohjavirta, Chap. 10.1007/978-981-15-7018-6_25). Similarly, the massive open online course providers are innovating to create a robust lifelong learning ecosystem to upskill and reskill the workforce around the globe with micro-credentialing of curated courses that continuously respond to emerging labor market needs (Qiu, Chap. 10.1007/978-981-15-7018-6_26).

With the growing demand for workers with information and technology (IT) knowledge and skills, many countries are requiring their education systems to include coding from an early stage to prepare students for future jobs in IT (Nambiar, Chap. 10.1007/978-981-15-7018-6_28). In India where a large percentage of engineers joining the IT industry do not come with the expected skills to work in customer projects, there is a big demand to train them so they are able to understand systems, solve problems, communicate effectively with customers, and relate to real-world problems (Parthasarathy, Chap. 10.1007/978-981-15-7018-6_27). To address the acute problem of a lack of qualified teachers in many developing countries, online blended programs are emerging as an effective way of promoting teacher professional development programs at scale without losing the quality that cascade training face (Lim et al, Chap. 10.1007/978-981-15-7018-6_28).

**6. Technology platforms can reduce skills mismatches by better anticipating and preparing for emerging skills and jobs (Part VI)**. In the current age of digital disruptions, the nature of work is changing rapidly, with a growing demand for higher order skills and decreasing demand for lower order, routine skills that can be automated. While the technologies that emerged from previous industrial revolutions also displaced workers using manual labor, the question now is, is it different this time? (Khatiwada, Chap. 10.1007/978-981-15-7018-6_32) Singapore is reducing skills mismatches by developing real-time labor market information systems using artificial intelligence and big data analytics to help job seekers match their skills profiles with the skills that potential employers are seeking (Gan, Chap. 10.1007/978-981-15-7018-6_31). In the ROK, there are continuous efforts to enhance collaboration between skills development and public employment services to reduce skills mismatches using a combination of different approaches: (i) regular labor demand surveys, (ii) long-term labor demand surveys, and (iii) AI and big data analytics to replace traditional approaches to promote job-matching (Lee, Chap. 10.1007/978-981-15-7018-6_33).

Another important side is how schools and training providers are responding to 4IR challenges in reducing skills mismatches. Due to a lack of information, access, and guidance, a large percentage of Filipino youth are not entering higher education. To reverse this trend, and to capitalize on the digital exposure of Gen Z (those borne from 1995 onward), it is possible to provide online information on schools, courses, and careers to encourage them to acquire higher level skills (Motte-Muñoz, Chap. 10.1007/978-981-15-7018-6_30). There is a need for training institutions to focus on the regular renewal of curriculum to match with emerging industry needs and to foster close collaboration between government, industry, and training providers to support lifelong learning for continuous reskilling and upskilling of the existing workforce.

Finally, according to UNESCO, around 1.5 billion students were out of school in over 180 countries due to the COVID-19 pandemic. In some countries like the People’s Republic of China, online learning has surged and, for the first time, this also forced public education to seek online learning at an unprecedented scale. Many governments are now more open to online learning and EdTech solutions, which will require rethinking education, promoting innovations, and developing evidence for improving teaching and learning. Drawing lessons from such experiences will be important to building on this momentum to improve the quality of education particularly for those students that are lagging behind. This will also require strong coordination and partnerships to develop a flexible learning management system that can interface with some of the best available multi-channel platforms (online, Offline, TV and radio, delivery of printed materials), experts, and teachers and trainers.

**7. Education and training is a major catalyst that can bust silos and promote cross-sectoral collaboration for sustainable economic transformation in developing countries (Part VII)**. Education is key to unleash the full potential of human capital since it provides the foundation for gender equality, healthy lifestyles, employability, innovation, peace, and sustainability. Therefore, education is seen as a catalyst, and the *master key*, to achieve all the 17 sustainable development goals (SDGs). Education is a prominent component of the global competitive index (interrelationship between education, skills, and work), as it is for the global innovation index (education’s close relationship with skills, entrepreneurship, science & technology, and research & development).

Part VII highlights five interrelated examples of how education drives cross-sectoral collaboration. First, the example of a pilot in Mongolia (Build4Skills) shows how TVET can be integrated with infrastructure projects to drive workforce development through on-the-job training in partnership with industry (Edel, Chap. 10.1007/978-981-15-7018-6_35). Such an approach provides excellent opportunities to companies to not only improve the quality of infrastructure but also help develop skills of the workforce, particularly in countries that rely on foreign labor.

Second, the example of how a university can use STEAM (science, technology, engineering, arts, and mathematics) education to prepare youth with employability as well as entrepreneurship skills in Thailand demonstrates how twenty-first-century skills can be developed while promoting sustainable development (Liu, Chap. 10.1007/978-981-15-7018-6_36).

Third, the example from Tajikistan on how TVET can train women in non-traditional, higher paying technical fields and occupations demonstrates a promising strategy to provide more inclusive TVET to women (Izawa, Chap. 10.1007/978-981-15-7018-6_37). This approach promoted the combination of social marketing, campaigns, stipends, dormitories, and female-friendly facilities. Fourth, the example of the Torino Process promoted by the European Training Foundation shows how a combination of traditional and nontraditional approaches can make TVET more inclusive while addressing the different needs of diverse students (Onestini, Chap. 10.1007/978-981-15-7018-6_38).

Finally, in light of the massive disruption of IR4 on the future of skills and jobs, there is a need to reskill and upskill the existing workforce in a continuous manner as part of lifelong learning. Singapore’s SkillsFuture demonstrates how this can be done through a voucher scheme that incentivizes individuals and companies to reskill and upskill through a choice of highly relevant courses offered by national and international training providers through blended learning platforms (Fung, Chap. 10.1007/978-981-15-7018-6_39). Such an approach is gaining traction everywhere, as a result of which reskilling and upskilling is developing into one of the largest industries in the world.

The future world of work requires agile and independent workers who are ICT literate and readily able to upskill and/or retool. To prepare for this future, higher education and TVET systems need to be conducive for lifelong learning, enabled by various learning and training modalities of skills development. At the same time, national qualifications systems, learning pathways, and recognition and validation systems of learning also need to be enhanced to motivate learners and workers to continuously learn. Linkages among governments, industries, and educational institutions would be critical in ensuring that curricula and learning interventions are relevant. Finally, development cooperation would be instrumental in addressing learning inequities among regions and countries as well as in sharing good practices and replicating effective models for lifelong learning through continued investment in human capital development.

## Footnotes

1 Asian Development Bank. 2020. Asia’s Journey to Prosperity: Policy, Market and Technology Over 50 Years.

2 Ibid.

3 McKinsey & Company. 2007. How the world’s best-performing school systems come out on the top.

4 World Bank. 2018. World Development Report: Learning to Realize Education’s Promise.

5 Manpower Group. 2015. 2015 Talent Shortage Survey.

6 Asian Development Bank. Forthcoming (2020). Assessing skills and technology for high-growth industries in South-East Asia: Insights from Cambodia, Indonesia, the Philippines, and Viet Nam

7 Howard Gardner. 2011. Frames of Mind: The Theory of Multiple Intelligences. Basic Books.

8 Todd Rose. 2016. The End of Average. Harper and Collins Publishers.

9 Manos Antoninis and Silvia Montoya. July 2019. The World is Off Track to Deliver on its Education Commitments by 2030.

10 The International Commission on Financing Global Education Opportunity. 2016. The Learning Generation: Investing in Education for a Changing World.

11 World Bank. 2018. World Development Report: Learning to Realize Education’s Promise.

12 There are a number of research findings that show the potential impact of EdTech solutions on learning outcomes: (i) J-PAL. 2016. Will technology transform education for the better?; (ii) Karthik Muralidharan et al. 2016. Disrupting Education? Experimental Evidence on Technology-Aided Instruction in India. National Bureau of Economic Research Working Paper 22923; (iii) Maya Escueta et al. 2017. Education Technology: An Evidence-Based Review. National Bureau of Economic Research Working Paper 23744; (iv) Rob Sampson et al. 2019. The EdTech Lab Series: Insights from rapid evaluations of EdTech products. Central Square Foundation and ID Insight.

## References

[CR1] Antoninis, M., & Montonya, S. (2019). The world is off track to deliver on its education commitments by 2030.

[CR2] Asian Development Bank. (2020a). Asia’s journey to prosperity: Policy, market and technology Over 50 Years.

[CR3] Asian Development Bank. (2020b, Forthcoming). Assessing skills and technology for high-growth industries in South-East Asia: Insights from Cambodia, Indonesia, the Philippines, and Vietnam.

[CR4] Escueta, M., et al. (2017). Education technology: An Evidence-Based review. National Bureau of Economic Research Working Paper 23744.

[CR5] Gardner, H. (2011). *Frames of mind: The theory of multiple intelligences*. Basic Books.

[CR6] J-PAL. (2016). Will technology transform education for the better?

[CR7] Manpower Group. (2015). 2015 Talent Shortage Survey.

[CR8] McKinsey & Company. (2007). How the world’s best performing school systems come out on the top.

[CR9] Muralidharan, K., et al. (2016). Disrupting education? Experimental evidence on technology-aided instruction in India. National Bureau of Economic Research Working Paper 22923.

[CR10] Rose, T. (2016). *The end of average*. Harper and Collins Publishers.

[CR11] Sampson, R., et al. (2019). The EdTech lab series: Insights from rapid evaluations of EdTech products. Central Square Foundation and ID Insight.

[CR12] The International Commission on Financing Global Education Opportunity. (2016). The learning generation: Investing in education for a changing world.

[CR13] World Bank. (2018). World development report: Learning to realize education’s promise.

